# The Effect of Ethanol on Lipid Nanoparticle Stabilization from a Molecular Dynamics Simulation Perspective

**DOI:** 10.3390/molecules28124836

**Published:** 2023-06-17

**Authors:** Ari Hardianto, Zahra Silmi Muscifa, Wahyu Widayat, Muhammad Yusuf, Toto Subroto

**Affiliations:** 1 Department of Chemistry, Faculty of Mathematics and Natural Sciences, Universitas Padjadjaran, Jatinangor 45363, West Java, Indonesia; 2 Research Center for Molecular Biotechnology and Bioinformatics, Universitas Padjadjaran, Bandung 40133, West Java, Indonesia; 3 Faculty of Pharmacy, Mulawarman University, Samarinda 75119, East Kalimantan, Indonesia

**Keywords:** ethanol, lipid nanoparticle, molecular dynamics simulation, stability

## Abstract

Lipid nanoparticles (LNPs) have emerged as a promising delivery system, particularly for genetic therapies and vaccines. LNP formation requires a specific mixture of nucleic acid in a buffered solution and lipid components in ethanol. Ethanol acts as a lipid solvent, aiding the formation of the nanoparticle’s core, but its presence can also affect LNP stability. In this study, we used molecular dynamics (MD) simulations to investigate the physicochemical effect of ethanol on LNPs and gain a dynamic understanding of its impact on the overall structure and stability of LNPs. Our results demonstrate that ethanol destabilizes LNP structure over time, indicated by increased root mean square deviation (RMSD) values. Changes in the solvent-accessible surface area (SASA), electron density, and radial distribution function (RDF) also suggest that ethanol affects LNP stability. Furthermore, our H-bond profile analysis shows that ethanol penetrates the LNP earlier than water. These findings emphasize the importance of immediate ethanol removal in lipid-based systems during LNP production to ensure stability.

## 1. Introduction

Genetic therapies derived from interfering RNA (siRNA), mRNA, and plasmid DNA can potentially treat many diseases [1]. However, the immune system can rapidly degrade naked DNA and RNA. Encapsulating DNA or RNA molecules into lipid nanoparticles (LNPs) is a method of overcoming this problem [2]. Encapsulating RNA into LNPs also prevents cleavage by RNase in the blood [3]. Research has shown that LNPs effectively transport genetic material into target cells, both for siRNA and mRNA [4]. Furthermore, LNP is a key technology behind the success stories of the Pfizer-BioNTech and Moderna COVID-19 vaccines [5].

The LNP structure usually consists of ionizable lipids, structural lipids, and cholesterol. The ionizable lipids have positive charges at low pH and are neutral at the physiological pH. This characteristic of ionizable lipids increases LNP biocompatibility, which is beneficial for genetic drug delivery [2]. Structural lipids play a role in the stability of LNPs, while cholesterol is involved in the intracellular delivery of LNPs [6]. Moreover, cholesterol can maintain the integrity of the LNP structure [7]. LNPs also contain a small amount of DMG-PEG2000 to prevent aggregation [8].

LNPs were produced by combining nucleic acid with an acidic aqueous buffer and lipid excipients in ethanol, which has the advantage of low toxicity compared to other lipid solvents [9]. Once the LNPs are formed, ethanol must be removed promptly to maintain the integrity of the LNPs. A study by Kimura and coworkers [10] has revealed that post-treatment with ethanol dialysis can stabilize the LNP structure since ethanol can destabilize the lipid membrane. However, the mechanism at the molecular level remains to be determined, and research related to the molecular mechanism of LNPs requires extensive resources and time.

MD simulations have been used to investigate the behavior of lipid nanoparticles (LNPs) in drug delivery applications, emphasizing their potential for optimizing LNP designs [11]. Extensive investigations using MD simulations have also elucidated the self-assembly process of lipid bilayer complexes and the nanoparticles, offering insights into underlying mechanisms, driving forces, and structural and optical properties [12]. MD simulations can also reveal the pH-dependent behavior of lipids in the design of mRNA delivery systems. These findings significantly advance our understanding of lipid-based drug delivery systems [13]. 

In this study, we used molecular dynamics (MD) simulations to gain insight into molecular interactions and environmental systems from a physicochemical perspective regarding the effect of ethanol on LNP stability. We used SM-102 as the ionizable lipid, whereas DSPC was the structural lipid [14]. DMG-PEG2000 was not involved in the LNP model since we only simulated one LNP. By modeling the interactions between atoms and molecules over time, MD simulations can provide valuable understandings into the behavior of LNPs in the presence of ethanol. The resulting information has important implications for developing genetic vaccines and drug delivery platforms. 

## 2. Result

### 2.1. Molecular Dynamics Simulations of Lipid Nanoparticle Self-Assembly

Before investigating the effect of ethanol on the stabilization of LNPs using MD simulation, we prepared the LNP model. In the wet laboratory, the formation of LNPs begins with mixing nucleic acid in an acidic aqueous buffer and lipid excipients in ethanol solvent, followed by dialysis leading to the precipitation and self-assembly of the particles [15]. Lipid molecules tend to self-assemble into LNPs to reduce the exposure of their hydrophobic regions to water and maximize hydrogen bonding interactions between the nucleosides of the siRNA [16]. Therefore, we only used water as the solvent for the self-assembly MD simulations to accelerate LNP formation [17]. 

**Figure 1:**
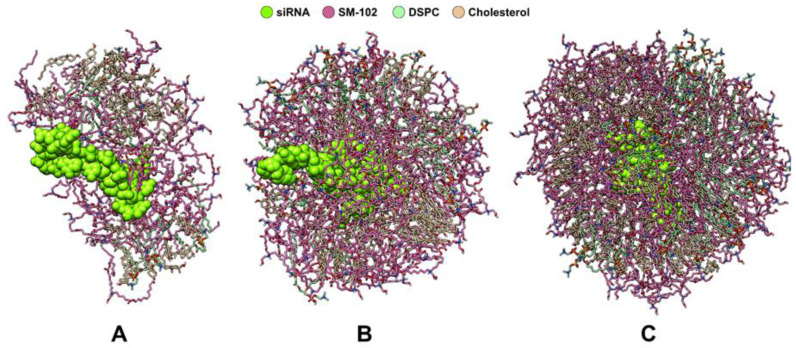
MD simulation trials of LNP self-assembly using N/P ratios of (**A**) 5:2, (**B**) 10:2, and (**C**) 15:2. siRNA is shown as a chartreuse VdW representation, while lipids are shown as stick representations: the hot pink color is SM-102, the light green color is DSPC, whereas the khaki color is cholesterol.

The N/P ratio in the lipid nanoparticle system refers to the ratio of the number of the positively charged amine group (N) in SM-102 to the number of the negatively charged phosphate group (P) in siRNA. For instance, the N/P of 5:2 means that there are 50 SM-102 particles and one siRNA molecule consisting of 21 base pairs, where each SM-102 molecule possesses one amine group, while each base pair has one phosphate group. We started with an N/P ratio of 5:2 which contains one siRNA molecule and lipids consisting of 50 SM-102, 39 cholesterol, and 10 DSPC molecules (Table 1); such numbers are based on the literature [14]. The system was modeled using packmol and tleap programs. Subsequently, we subjected the model to a 100 ns MD simulation. However, the result (Figure 1A) reveals that lipid molecules do not fully cover the siRNA molecule surface. Therefore, in the second MD simulation, we doubled the number of lipid molecules to achieve an N/P ratio of 10:2 (Table 1). The result (Figure 1B) shows that more lipid molecules are required to encapsulate siRNA thoroughly. Hence, we performed the third MD simulation with an N/P ratio of 15:2, which contains 150 SM-102, 120 cholesterol, and 30 DSPC molecules (Table 1). The last MD simulation gives an LNP, which fully covers siRNA, as shown in Figure 1C. 

**Figure 2:**
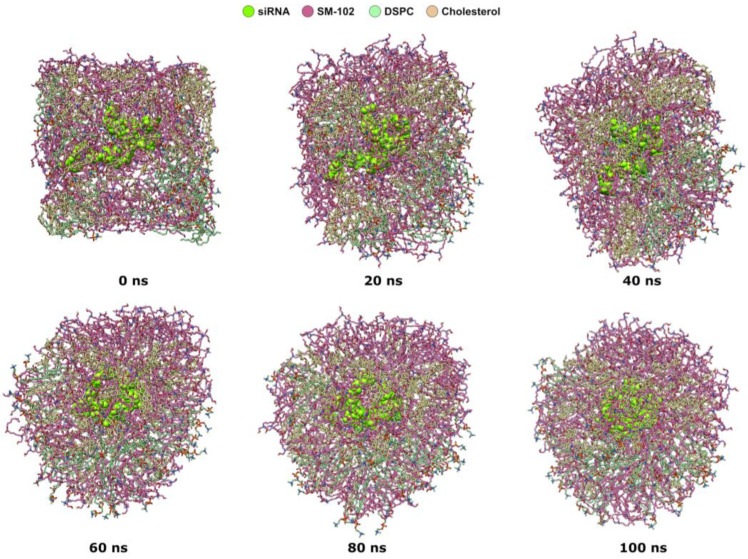
MD trajectory snapshots of LNP self-assembly with an N/P of 15:2 in the first 100 ns. The snapshots were taken every 20 ns. siRNA is shown as a chartreuse VdW representation, while lipids are shown as stick representations: the hot pink color is SM-102, the light green color is DSPC, whereas the khaki color is cholesterol.

The formation LNP with an N/P ratio of 15:2 occurs quickly before 100 ns (Figure 2). At 0 ns of the MD production stage, the siRNA molecule is at the center of the periodic boundary box and surrounded by lipid molecules. As the simulation runs, lipid molecules around siRNA gradually form a spherical shape from the cubic one. Based on MD trajectory snapshots every 20 ns in the first 100 ns, the transformation of lipid molecules around siRNA is noticeable at 20 ns. At 60 ns, the lipid molecules around siRNA arrange themselves. The spherical shape becomes clear at 60 ns and is more compact at 100 ns.

In a more detailed analysis (Figure S1 in Supplementary Materials), some SM-102 molecules directly interact with siRNA, covering the nucleic acid on the first layer. Meanwhile, the other SM-102 molecules are on the second layer, like most cholesterol and DSPC molecules (Figure S1). The primary interaction formed by SM-102 and siRNA is the electrostatic interaction, as depicted in Figure S2A. Electrostatic interactions between the negatively charged nucleic acid (from the phosphate groups) and positively charged SM-102 (from the protonated amine group) result in the encapsulation of the nucleic acid [18]. Additionally, SM-102 molecules can interact with siRNA through hydrogen bonding (H-bond), non-conventional H-bond, and hydrophobic interactions (Figure S2B). The study by Wei Wang et al. [17] using MD simulation allowed the examination of the aggregation pattern and molecular composition of the LNPs. The study showed that the RMSD profiles of the four lipid systems reached a stable state after about 50 ns, which is similar to our result. In a separate study, Fernandez-Luengo et al. [11] observed that water molecules could diffuse across the lipid core of nanoparticles, allowing for the exchange of water between the interior and exterior of the LNP.

### 2.2. Molecular Dynamics Simulations of A Lipid Nanoparticle in an Aqueous Ethanol Environment

To investigate the impact of ethanol on LNP structure stability, a 1-μs MD simulation was performed in an aqueous solvent environment containing 24% ethanol. The ethanol concentration employed in the LNP system was chosen according to other research [14] and our laboratory work (unpublished results). The experimental parameters involved an ethanol-to-water ratio of 3:1. Based on these conditions, we calculated the ethanol concentration in the system to be 24%. The number of solvent particles is calculated based on the solute partial specific volume and the solvent concentration. The resulting 1-μs MD trajectory was subjected to the calculations of root mean square deviation (RMSD), hydrogen-bonding (H-bond) interactions, electron density, radial distribution function, and solvent accessible surface area (SASA).

#### 2.2.1. RMSD

**Figure 3:**
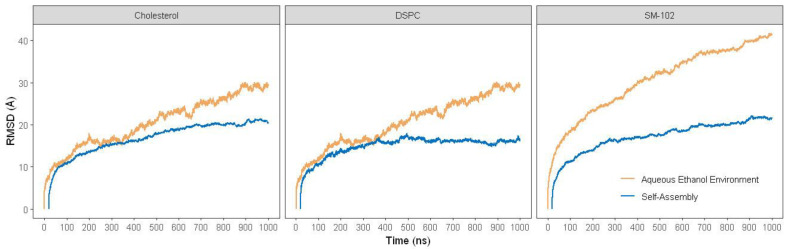
Root mean square deviation (RMSD) of LNP structure with N/P ratio of 15:2 over 1000 ns MD simulations of self-assembly and aqueous ethanol systems. The blue lines denote lipids in the self-assembly system, whereas the orange lines are lipids in the aqueous ethanol system.

Initially, we performed RMSD profile comparisons of all lipids in the self-assembly and aqueous ethanol systems over 1000 ns of MD trajectories, respectively (Figure 3). Since we found that, in the self-assembly system, the transformation of lipid molecule arrangement around siRNA is at 20 ns, we started RMSD calculation from the frame at 20 ns. 

The self-assembly system shows lower RMSD values than the aqueous ethanol system for all lipids (Figure 3). Additionally, all lipids in the self-assembly system show more stability trends than those in the aqueous ethanol system. The increased trends suggest that all lipids in the aqueous ethanol system experience considerable conformational changes, which may correlate to the integrity weakening of the LNP. 

#### 2.2.2. Solvent Accessible Surface Area (SASA)

**Figure 4:**
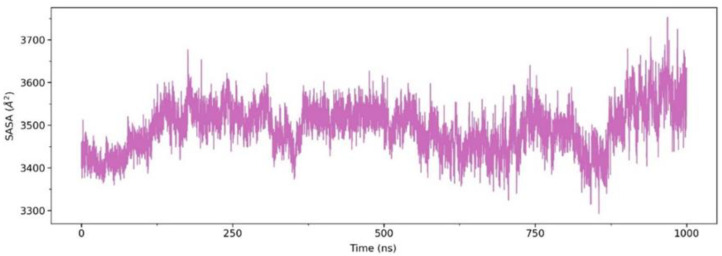
Solvent accessible surface area (SASA) of the siRNA structure in the aqueous ethanol system over 1000 ns.

According to the RMSD analysis above, the aqueous ethanol system may weaken the integrity of the LNP, where water and ethanol molecules may penetrate the LNP and affect the SASA of the siRNA. Therefore, we analyzed the SASA of the siRNA. As shown in Figure 4, the SASA values increase from around 3400 to 3600 Å^2^ near 130 ns. This observation may indicate the broken-down LNPs due to the aqueous ethanol environment, which causes the siRNA to be more exposed to solvent molecules. Based on this increase in SASA values, we suspected that the penetration of ethanol and water molecules into the LNPs starts before 100 ns. Subsequently, the SASA values of siRNA fluctuate until around 800 ns before increasing sharply to 3700 Å^2^ near 1000 ns. Such an increase in SASA values reflects a greater surface area of siRNA exposed to solvents, which may indicate structural damage to the LNP.

#### 2.2.3. Electron Density Profiles (EDPs)

**Figure 5:**
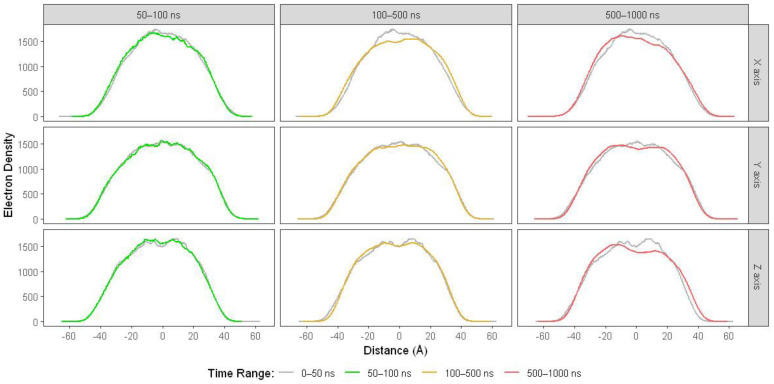
EDPs of the lipid components in the aqueous ethanol system over 1000 ns. The grey lines represent EDPs from 0 to 50 ns, the green lines are EDPs from 50 to 100 ns, whereas the orange and red lines denote EDPs from 100 to 500 ns and 500 to 1000 ns, respectively.

Penetration of water and ethanol molecules into the LNP may cause gaps between lipids, which may be detected by EDPs. Thus, we carried out electron density calculations on the lipids. We compared EDPs of lipids at 0–50 ns with the ones at 50–100, 100–500, and 500–1000 ns. The results (Figure 5) reveal that the EDP at 50–100 ns, on the x-axis, is lower than that of 0–50 ns. Such an EDP decrease becomes noticeable at 100–500 ns. At 500–1000 ns, EDPs decrease more evidently on all axes, which may indicate significant structural damage to the LNPs.

Moreover, we conducted EDP calculations on ethanol as an alternative perspective. As shown in Figure S3, EDPs of ethanol increase around the middle on all axes during MD simulations. These results suggest that ethanol molecules penetrate the LNP and surround the siRNA molecule. We also found similar results for EDPs of water (Figure S4), although they were not too noticeable. All EDPs (Figure 5 and Figures S3 and S4) suggest that water and ethanol molecules penetrate the LNPs before 100 ns, as shown in the comparison at 0–50 and 50–100 ns. These results also reveal that penetration may occur earlier, before 50 ns. 

#### 2.2.4. Radial Distribution Function (RDF)

**Figure 6:**
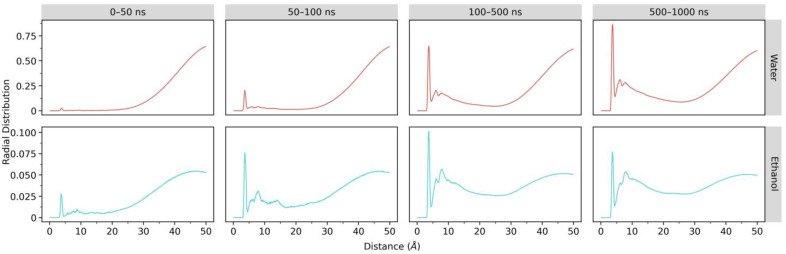
Radial distribution functions (RDFs) of ethanol and water with P-siRNA atom as the reference atom in the aqueous ethanol system over 1000 ns. The red lines are RDFs between water and siRNA, whereas the turquoise lines denote RDFs between ethanol and siRNA.

To further clarify our notion that ethanol and water molecules begin to penetrate the LNPs under 50 ns of MD simulation, we utilized RDF analysis which can portray the probability of finding a particle at a given distance from a reference in a system [19]. We used phosphor atoms as the probe for siRNA and oxygen atoms for both ethanol and water in the RDF calculations within four-time intervals of 0–50, 50–100, 100–500, and 500–1000 ns. RDF analysis results (Figure 6) support our notion. Some portions of both solvent molecules are already less than 5 Å away from siRNA within a 0–50 ns interval. In the next time intervals, the RDF values below 5 Å rise for water and ethanol, where the highest density for ethanol is in the time range of 100–500 ns, while for water, the highest is within 500–1000 ns. Furthermore, RDF values between 5 and 15 Å increase for both solvents during the MD simulation. These results also suggest that the aqueous ethanol environment causes structural damage to the LNP.

**Figure 7:**
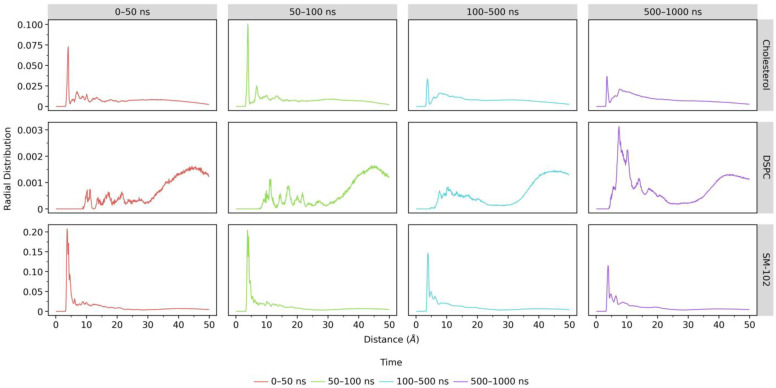
RDFs of lipid molecules, with P-siRNA atom as the reference atom, in the aqueous ethanol system over four-time ranges of MD simulation. The red lines represent RDFs from 0 to 50 ns, the green lines denote RDF from 50 to 100 ns, whereas the turquoise and purple lines are RDFs from 100 to 500 ns and 500 to 1000 ns, respectively.

We also carried out RDF analyses between lipids and siRNA. Nitrogen atoms were employed as the probes for SM-102 and DSPC, while oxygen atoms were employed for cholesterol and phosphor atoms for siRNA. As displayed in Figure 7, RDF values below 5 Å for SM-102 decrease over the simulation time, whereas the values between 5 and 10 Å increase. These results suggest that SM-102 molecules, the major lipid component of LNPs, move away from siRNA. A similar pattern is also observed on RDF cholesterol profiles. Meanwhile, DSPC shows an increase in RDF values between 5 and 10 Å. These changes in RDF profiles of all lipids may indicate that the integrity of LNP is violated due to the aqueous ethanol environment. 

#### 2.2.5. Hydrogen Bonding (H-Bond)

**Figure 8:**
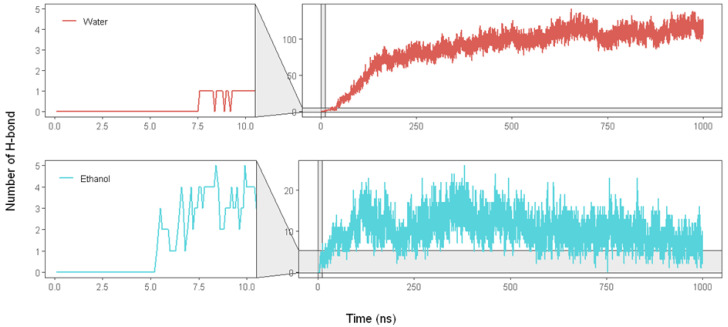
Number of hydrogen bonds formed between siRNA and solvents (water and ethanol) in the aqueous ethanol system over 1000 ns. The red lines are the number of hydrogen bonds between water and siRNA, whereas the turquoise lines denote those between ethanol and siRNA.

We also performed hydrogen bonding (H-bond) analysis between solvents and siRNA to investigate further the influence of the aqueous ethanol environment on the LNP. The visualization of the H-bond number (Figure 8) reveals that ethanol and water molecules quickly penetrate the LNP and interact with siRNA at the beginning of the MD simulation. 

Interestingly, ethanol molecules penetrate the LNP earlier than water molecules. Our simulation shows that ethanol forms H-bonds with siRNA at 5.3 ns, and with water at 7.6 ns. In the beginning, ethanol molecules display an increased H-bond number with siRNA to around 20 interactions, followed by a decreasing trend of such interaction until the end of the MD simulation. Meanwhile, water molecules show an increased H-bond number throughout the MD simulation. These results may indicate that ethanol molecules initiate LNP penetration and create channels for water molecules to enter. Subsequently, some water molecules replace ethanol molecules to interact with siRNA. 

#### 2.2.6. Visualization Analysis

Sun and Sun (1985) reported that ethanol could disrupt delicate structures of lipid-based systems, such as lipid nanoparticles (LNPs). Ethanol can dissolve non-polar substances, such as lipids, by penetrating their structure and disrupting their interactions [20]. As a result, the LNP structure becomes less stable, as indicated by increased RMSD values during the simulation time (Figure 3). Ethanol can affect the balance of forces, such as van der Waals interactions, hydrogen bonding, and electrostatic interactions in LNP. Such disruptions weaken the stability of the LNP structure [21], as seen in shifts in SASA (Figure 4), EDP (Figure 5), RDF (Figure 6 and Figure 7), and H-bond (Figure 8) profiles. To obtain another insight into the effect of such organic solvent on the LNP, we visualized the interactions between ethanol and lipid molecules.

**Figure 9:**
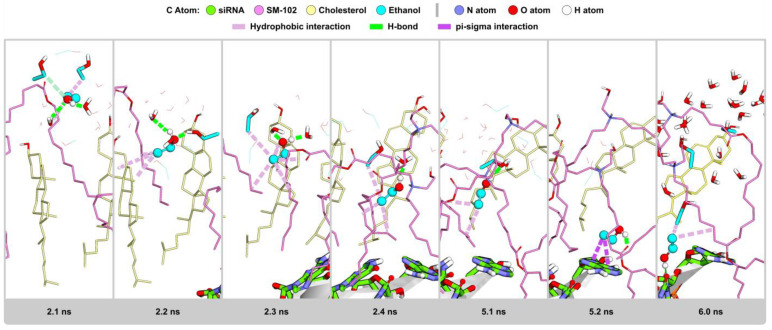
A representative snapshot of the entry solvent into the LNP structure. The C atoms of siRNA are in green and those of SM-102 are in hot pink; those of DSPC are in light green, whereas those of cholesterol and ethanol are in khaki and turquoise, respectively. The N atoms are in blue, while the oxygen and hydrogen atoms are in red and white, respectively. The pink dashed lines denote hydrophobic interactions, the green dashed lines are H-bonds, and the magenta dashed lines are pi–sigma interactions.

As shown in Figure S5, ethanol molecules surround the LNP surface at the beginning of the MD simulation. Initially, an ethanol molecule may interact with water and other ethanol molecules through H-bonds and hydrophobic interactions, respectively (Figure 9 at 2.1 ns). Subsequently, ethanol may form hydrophobic interactions with LNP components, such as cholesterol, using its ethyl moiety, as shown in Figure 9 at 2.2 ns. While its ethyl moiety points to the LNP core, its hydroxyl group faces outside and interacts with water and other ethanol molecules through H-bonds. At the following MD simulation time (Figure 9 at 2.2–5.1 ns), ethanol penetrates deeper into the LNP core using hydrophobic interactions with the surrounding lipids. The adjacent other ethanol may also interact with lipids (Figure S6), which may assist the first ethanol penetrating deeper into the LNP. Water and other ethanol molecules may follow the movement of the first ethanol (Figure 9 at 2.3–5.1 ns). As a result, water molecules can penetrate the LNP and eventually interact with siRNA (Figure 9 at 5.2 and 6.0 ns).

**Figure 10:**
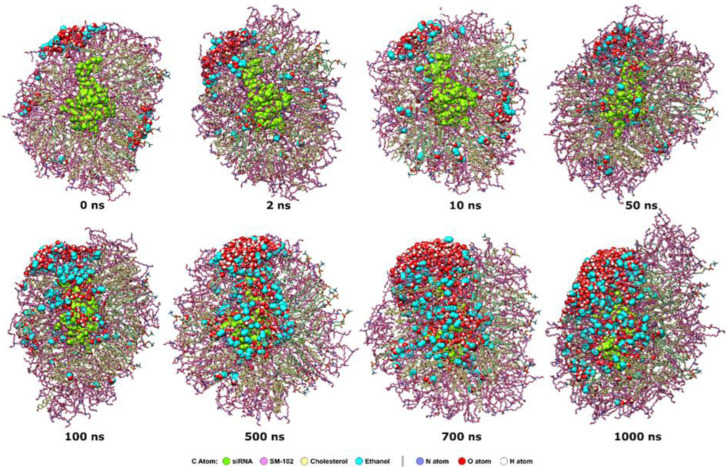
The effect of the presence of ethanol in the selected MD simulation times. siRNA is shown as a chartreuse VdW representation. Similarly, ethanol and water are also shown as VdW representations with different color codes. C atoms of ethanol are in turquoise, those of SM-102 are in hot pink, whereas those of DSPC and cholesterol are in light green and khaki, respectively. The N atoms are in blue, while the oxygen and hydrogen atoms are in red and white, respectively.

The interaction between ethanol and lipids involves the dissolution of lipids in ethanol due to its hydrophobic nature, which reduces lipid packaging and potential changes in lipid structure. Ethanol is less polar and has a lower dielectric constant than water, which makes it easy to break non-covalent interactions like hydrogen bonds or hydrophobic interactions [22]. Ethanol can act as a cosolvent, reducing the strength of the interactions between the lipids and causing the LNP structure to become less stable. Additionally, ethanol can also cause changes in the fluidity of the lipids, making the LNP more susceptible to environmental factors [23]. Thus, the presence of ethanol in lipid-based systems should be carefully considered in LNP production. Our MD simulation results of the aqueous ethanol system (Figure 10) show that the number of ethanol molecules penetrating the LNP structure increases over time, causing structural damage to the LNP.

## 3. Materials and Methods

### 3.1. Force Fields

The lipids utilized in this study, including DSPC and cholesterol, were sourced from the Amber lipid force fields, LIPID21 [24]. For SM-102, we used Austin model 1—bond charge corrections (AM1-BCC) [25] through the antechamber program to compute the atomic charges of the ionic lipid. Other parameters for SM-102 were assigned from General Amber Force-fields 2 (GAFF2) [26], while the additional parameters were determined using the parmchk program in AmberTools19 [27]. Parameterization for ethanol followed the same steps as SM-102. The optimized structure of ethanol was obtained from the Automatic Topology Builder [28]. We used the TIP3P water model [29] for water molecules, whereas the OL3 parameters [29] were for the siRNA

### 3.2. Molecular Dynamics Simulations

In the early stage, we performed MD simulations to obtain a well-encapsulated LNP structure. We utilized a siRNA consisting of 21 base pairs: 5′-GCAACAGUUACUGCGACGUUU-3′ [30]. We selected this siRNA due to its relatively small structure, reducing computation time. The simulation systems were prepared using molar ratios of SM-102 to cholesterol to DSPC, as tabulated in Table 1. All lipid systems were randomly placed in a box with dimensions of 80 Å × 80 Å × 80 Å, whereas siRNA was placed at the center of the box using the packmol program [31]. The subsequent preparation processes used the tleap program in AmberTools20 [32] for water solvation and to yield topology and coordinate files. 

The topology and coordinate files were subjected to a 50.000-step minimization, with a 500 kcal/mol Å constraint. Every system was then heated gradually to 300 K for 10 ps. In the following step, every system was equilibrated, using a Langevin protocol under a constant volume and temperature (NVT) ensemble for 3 ps with a time step of 1 fs and a constraint of 5 kcal/mol Å. The production steps were run to yield a 100-ns trajectory for each system using Particle-Mesh Ewald Molecular Dynamics (PMEMD) powered by a graphical processing unit implemented in Amber22 [32]. For the system completely encapsulating siRNA, we extended the MD simulation to 1000 ns.

The first simulation was conducted in 100% water solvent, aiming to form aggregates of LNPs. The simulation was performed three times with different N/P ratios. Initially, the system was simulated to obtain a structure that can be well encapsulated. The composition of the N/P ratio in each simulation system is presented in Table 1. The N/P ratio, which is the total number of ionizable lipid amine groups (N) to the total number of negatively charged nucleic acid phosphate groups (P), is often a parameter that can be optimized during LNP formation [14]. The N/P ratio is an essential physicochemical property in polymer-based gene delivery [33]. The value of the N/P ratio affects various properties of LNPs, such as net surface charge, size, and stability [34]

The LNP model, obtained from the previous MD simulation in water, was further simulated in 24% ethanol. We calculated the number of solvent particles based on the solute partial specific volume and the solvent’s concentration. The system was subjected to an MD simulation with the same minimization, heating, and equilibration steps as the MD simulations for LNP self-assembly. The MD simulation in an aqueous ethanol environment produced a 1000 ns trajectory.

### 3.3. Simulation Analyses

To elucidate MD trajectory data, we used several analysis calculations provided in AmberTools19 [27]. The analyses are root mean square deviation (RMSD), solvent accessible surface area (SASA), electron density profiles (EDPs), radial distribution function (RDF), and hydrogen bonding (H-bond). 

RMSD evaluates the stability lipid system and the conformational changes during the simulation [35]. The RMSD values are calculated by translating and rotating the instantaneous structure coordinates to superimposed references with maximum overlap. RMSD is defined by Equation (1):

(1) $$RMSD=\sqrt{\frac{\sum_{i=1}^{N}m_{i}{\left|r_{i}-r_{i}^{ref}\right|}^{2}}{\sum_{i=1}^{N}m_{i}}}$$

where *m_i_* is the mass of atom *i*, whereas *r_i_* and *r_i_^ref^* are the coordinates of atom *i* at a particular instance during MD simulations and at its reference state. 

SASA analysis was applied to siRNA [36]. It was obtained by computing the Connolly surface area of the siRNA atoms. A sphere with a radius of 1.4 Å was used to represent the solvent molecule, which was then rolled over the siRNA molecule to produce a uniform and continuous contour for the outer surface.

EDPs give measurements of the average density of electrons across the LNP over a period of time. The EDPs were determined by assuming that an electron charge equal to the difference between the atomic number and the partial atomic charge was positioned at the center of each atom [37]. EDPs were computed using slices of 0.25 Å.

The radial distribution function (RDF) is the probability density of finding a particle at a distance *r* from the reference particle. The RDF is computed using a normalized histogram of particle count as a function of distance *r*. The normalization is as follows (Equation (2)):

(2) $$\mathrm{RDF}=\rho \;\frac{4\pi }{3}\left({\left(r+dr\right)}^{3}-r^{3}\right)$$

where *dr* is the bin spacing, and *ρ* is the density with a default value of 0.033456 molecules Å3, corresponding to a 1.0 g/mL water density [32].

Hydrogen bond analysis was carried out in solvents and siRNA structures with the default cut-off distance being less than 3.0 Å and an angle of 135° for Hydrogen bonds (H bonds) between donor and acceptor hydrogen [38].

All analyses were computed from the MD trajectories using the cpptraj program, as implemented in AmberTools20.

### 3.4. Molecular Visualization and Graph Generations

We utilized UCSF Chimera 1.15 [39] to visualize the whole picture of LNP, whereas Discovery Studio Visualizer v21.10.020298 (Dassault Systèmes, San Diego, CA, USA) was used to visualize the detailed molecular interactions. Graphs were generated using tidyr [40], ggplot2 [41], ggforce [41], gridExtra [42], ggpubr [43], and patchwork [44] packages on Jupyter Notebook 6.4.7 [45] (Project Jupyter, Berkeley, CA, USA) under an R programming language environment version 3.6.1 (R Foundation for Statistical Computing, Vienna, Austria) [46]. Artworks were created using Inkscape 1.1.1 [47] (The Inkscape Project, Boston, MA, USA).

## 4. Conclusions

In this study, we have conducted a 1 μs MD simulation of LNP in an aqueous solvent containing ethanol 24% (*v/v*). The RMSD analyses on SM-102, cholesterol, and DSPC indicate the destabilization of LNPs in ethanol aqueous solvent. SASA, EDP, and RDF analyses also collectively indicate LNP destabilization. Furthermore, H-bond analysis shows that ethanol penetrates LNP earlier than water. Meanwhile, visual inspection uncovers that hydrophobic interactions facilitate ethanol penetration to LNP: the ethyl moiety of ethanol interacts with non-polar parts of SM-102, cholesterol, or DSPC. The visual analysis also shows that ethanol breaks ground for water to penetrate LNP. Interestingly, our results pave the way for future MD simulation studies investigating the limit of ethanol tolerable by LNP. Another prospective study involves MD simulations of LNPs containing PEG lipid in an aqueous ethanol system. The presence of PEG lipid may influence ethanol’s effect on LNP stability since the lipid shields the LNP’s surface charge by creating a hydrophilic shell. In conclusion, this study has shed light on our understanding at a molecular level of the impact of ethanol on LNP destabilization and sparks other future investigations, with implications for lipid-based delivery system development.

## Figures and Tables

**Table 1:** Molecule number in three LNP simulation systems.

Parameter	N/P (5:2)	N/P (10:2)	N/P (15:2)
siRNA	1	1	1
SM-102	50	100	150
Cholesterol	39	80	120
DSPC	10	20	30
Water	11,352	67,358	75,800

## Data Availability

Data can be found in this article and the Supplementary Materials.

## Supplementary Materials

The following supporting information can be downloaded at: https://www.mdpi.com/article/10.3390/molecules28124836/s1. Figure S1: The arrangement every lipid component in LNP resulted by the MD simulation of the self-assembly system with N/P 15:2; Figure S2: Intermolecular interactions between siRNA and SM-102 lipid; Figure S3: Electron density profiles of the ethanol in the LNPs system for 1000 ns of MD simulation; Figure S4: Electron density profiles of the water in the LNPs system for 1000 ns of MD simulation; Figure S5: Visualization of ethanol molecules around LNP within 5 Å; Figure S6: Intermolecular interactions among ethanol, lipid, and water molecules.
