# Supplementary material for: The Effect of Ethanol on Lipid Nanoparticle Stabilization from a Molecular Dynamics Simulation Perspective
Source: Molecules. 2023 Jun 17;28(12):4836. doi: 10.3390/molecules28124836 (PMC10302858; doi:10.3390/molecules28124836)
Supplement: Supplementary file 1 [file molecules-28-04836-s001.zip › molecules-2413667-supplementary.pdf]

molecules

Article

The Effect of Ethanol on Lipid Nanoparticle Stabilization from a Molecular Dynamics Simulation Perspective

Ari Hardianto [1,2,*], Zahra Silmi Muscifa [2], Wahyu Widayat [2,3], Muhammad Yusuf [1,2] and Toto Subroto [1,2]

1 Department of Chemistry, Faculty of Mathematics and Natural Sciences, Universitas Padjadjaran, Jatinangor 45363, West Java, Indonesia; m.yusuf@unpad.ac.id (M.Y.); t.subroto@unpad.ac.id (T.S.)
2 Research Center for Molecular Biotechnology and Bioinformatics, Universitas Padjadjaran, Bandung 40133, West Java, Indonesia; zahra_silmi@apps.ipb.ac.id (Z.S.M.); wahyu@farmasi.unmul.ac.id (W.W.)
3 Faculty of Pharmacy, Mulawarman University, Samarinda 75119, East Kalimantan, Indonesia
* Correspondence: a.hardianto@unpad.ac.id

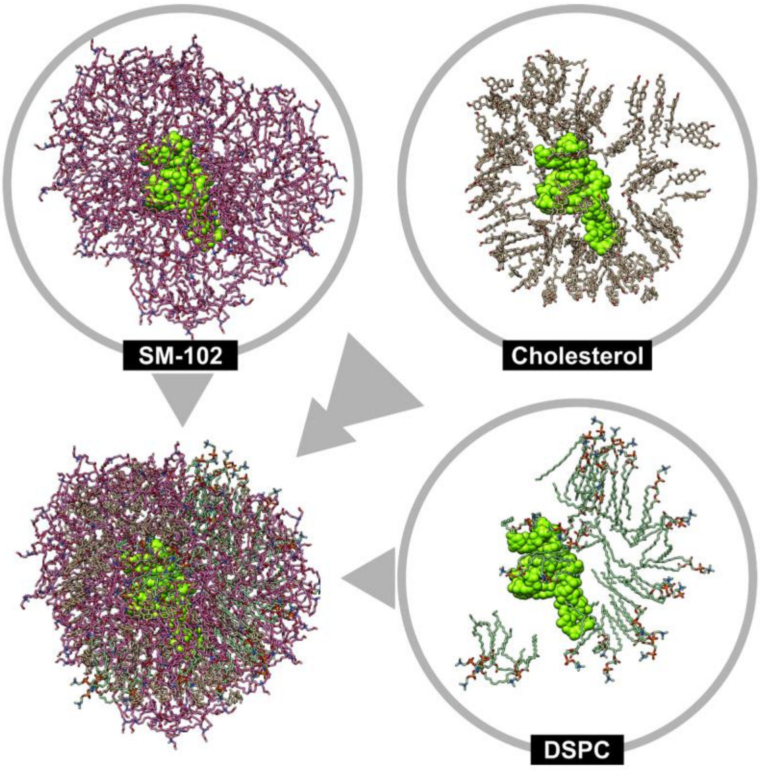

Figure S1. The arrangement every lipid component in LNP resulted by the MD simulation of the self-assembly system with N/P 15:2.

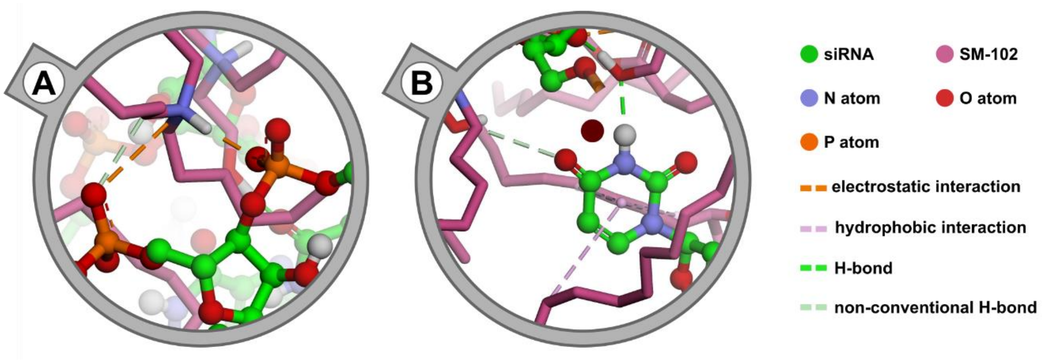

Figure S2. Intermolecular interactions between siRNA and SM-102 lipid.

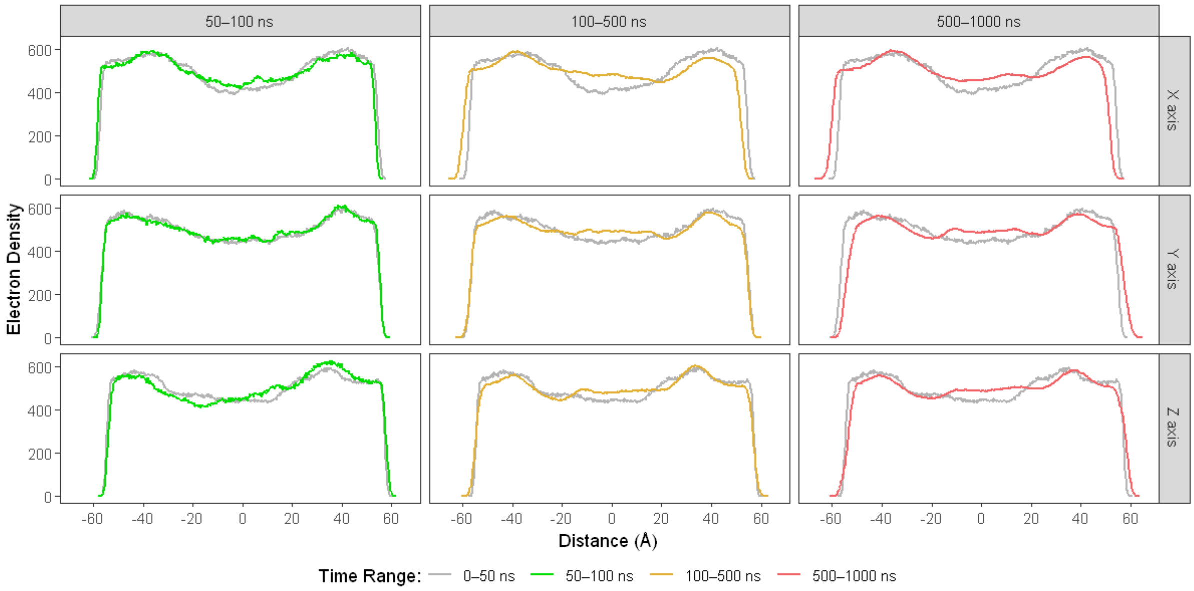

Figure S3. Electron density profiles of the ethanol in the LNPs system for 1000 ns of MD simulation.

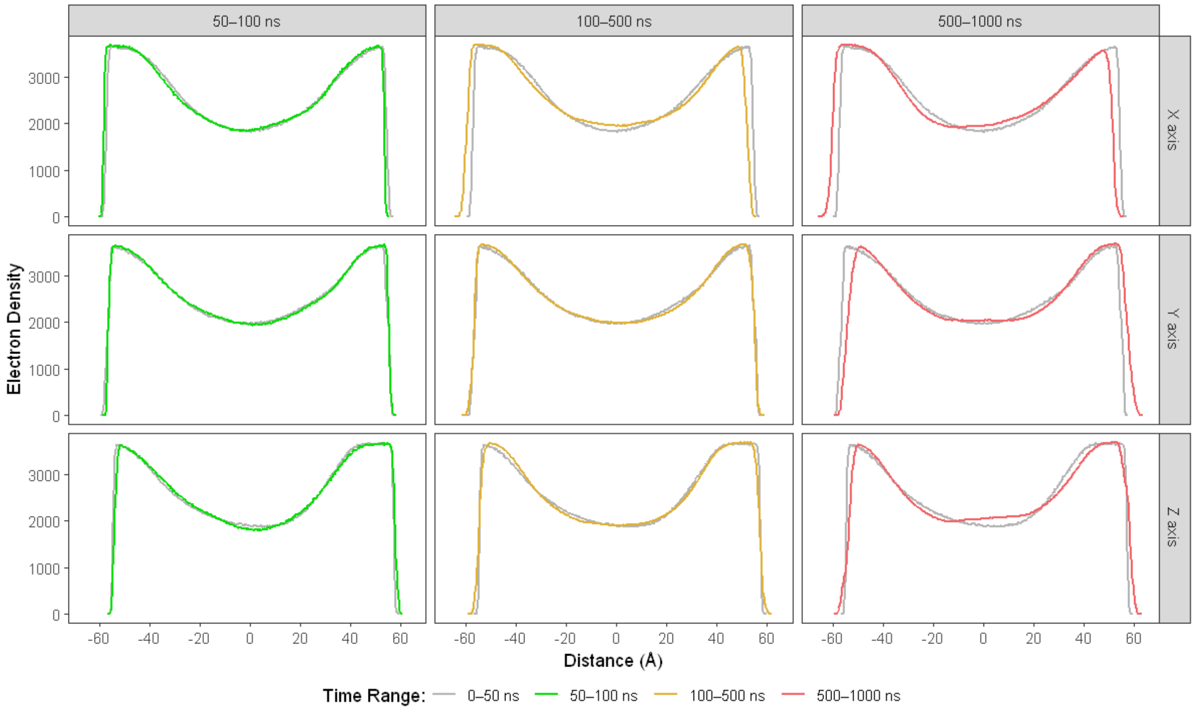

Figure S4. Electron density profiles of the water in the LNPs system for 1000 ns of MD simulation.

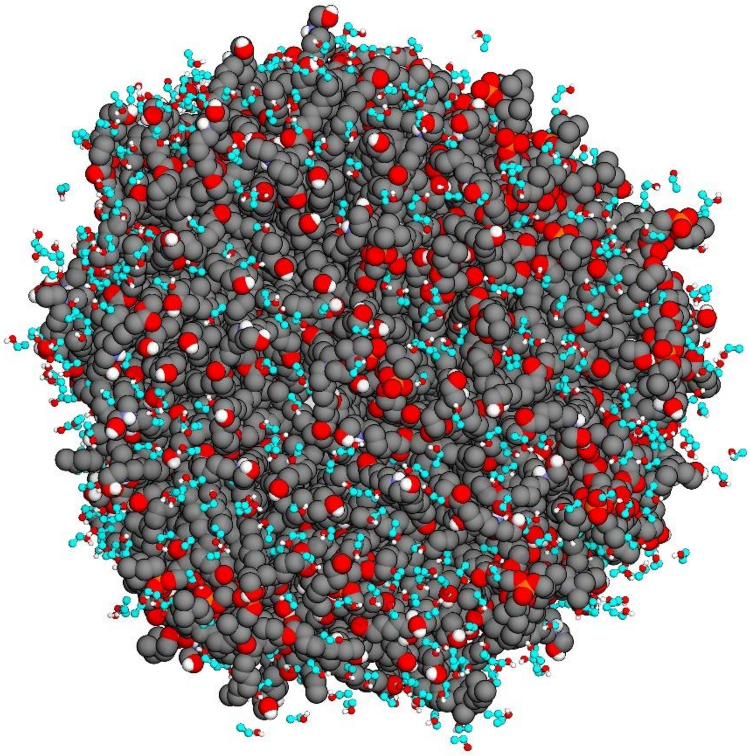

Figure S5. Visualization of ethanol molecules around LNP within 5 Å. Carbon atoms of ethanol molecules are in a cyan color.

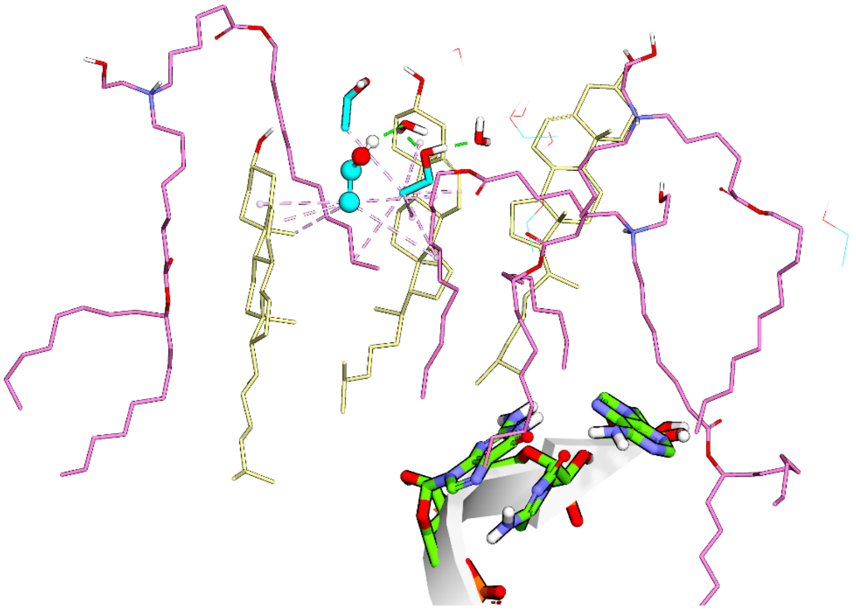

Figure S6. Intermolecular interactions among ethanol, lipid, and water molecules. Pink dashed lines indicate hydrophobic interactions, whereas the green ones are H-bonds.
